# Genome-Wide Identification and Characterization of Potato Long Non-coding RNAs Associated With *Phytophthora infestans* Resistance

**DOI:** 10.3389/fpls.2021.619062

**Published:** 2021-02-10

**Authors:** Weilin Cao, Liming Gan, Chenchen Wang, Xuechen Zhao, Mingyu Zhang, Jinwen Du, Shumei Zhou, Changxiang Zhu

**Affiliations:** State Key Laboratory of Crop Biology, College of Life Sciences, Shandong Agricultural University, Tai’an, China

**Keywords:** long non-coding RNA, *Solanum tuberosum* L., *Phytophthora infestans*, alternative splicing, TCseq, transiently overexpression

## Abstract

Long non-coding RNA (lncRNA) is a crucial regulatory mechanism in the plant response to biotic and abiotic stress. However, their roles in potato (*Solanum tuberosum* L.) resistance to *Phytophthora infestans* (*P. infestans*) largely remain unknown. In this study, we identify 2857 lncRNAs and 33,150 mRNAs of the potato from large-scale published RNA sequencing data. Characteristic analysis indicates a similar distribution pattern of lncRNAs and mRNAs on the potato chromosomes, and the mRNAs were longer and had more exons than lncRNAs. Identification of alternative splicing (AS) shows that there were a total of 2491 lncRNAs generated from AS and the highest frequency (46.49%) of alternative acceptors (AA). We performed R package TCseq to cluster 133 specific differentially expressed lncRNAs from resistance lines and found that the lncRNAs of cluster 2 were upregulated. The lncRNA targets were subject to KEGG pathway enrichment analysis, and the interactive network between lncRNAs and mRNAs was constructed by using GENIE3, a random forest machine learning algorithm. Transient overexpression of *StLNC0004* in *Nicotiana benthamiana* significantly suppresses *P. infestans* growth compared with a control, and the expression of extensin (*NbEXT*), the ortholog of the *StLNC0004* target gene, was significantly upregulated in the overexpression line. Together, these results suggest that lncRNAs play potential functional roles in the potato response to *P. infestans* infection.

## Introduction

The potato (*Solanum tuberosum* L.) is the fourth most important staple crop in global production after rice, maize, and wheat, and it is the most important non-grain crop for human consumption (Birch et al., 2012). However, multiple diseases can severely devastate the production and quality of potatoes, including late blight (Mizubuti and Fry, 2006), bacterial wilt (Wullings et al., 1998), and necrotic ringspot (Ohshima et al., 2000). The Irish famine of 1845–1852 caused by late blight led to around one million deaths and one million more people emigrated out of Ireland (Cantwell, 2017). Approximately billions of dollars are estimated as the cost of controlling the disease and damage caused by this disease per year (Haverkort et al., 2008).

Since the famine, potato late blight disease has attracted the attention of researchers, and more than 3,000 reports associated with the disease have been published (Fry et al., 2015). Control methods for the disease are divided into mainly two kinds: pesticide application and resistance breeding. The frequent use of systemic fungicides not only seriously pollutes the natural environment, but it also increases the probability of resistant populations by promoting the rapid evolution of numerous effector genes in the *Phytophthora infestans* genome (Raffaele et al., 2010). Therefore, breeding resistant varieties is the most effective control method. Investigating the mechanism of potato resistance to *P. infestans* plays an important role in the process of breeding resistant varieties.

Transcriptome sequencing is the main strategy for investigating the resistant mechanism at the genomic level. The differentially expressed genes associated with resistance pathways—salicylic acid-, jasmonic acid- (JA), and abscisic acid-mediated signaling pathways—were identified by sequencing the transcriptomes of potato infected by *P. infestans* at different stages (Duan et al., 2020). The hierarchies of resistance genes in the potato against late blight were investigated by integrating transcriptomic and metabolomic analysis (Yogendra and Kushalappa, 2016). The regulatory mechanism of miRNA in the tomato response to *P. infestans* was elucidated by high-throughput sequencing (Luan et al., 2015). Different resistance genes (*R* genes) and defense mechanisms against *P. infestans* infection between potato foliage and tuber were identified by the RNA-seq approach (Gao and Bradeen, 2016).

In addition to genes related to resistance pathways, *R* genes and miRNAs, long non-coding RNAs (lncRNAs) have also been proven to play an important role in gene expression and silencing pathways for several biological processes in response to pathogens (Zaynab et al., 2018). LncRNAs have no protein-coding ability, and their lengths are more than 200 nt (Sun et al., 2018). Based on their genomic location, lncRNAs are classified into three major categories, including long intergenic non-coding RNA (lincRNA), long non-coding natural antisense transcripts, and long intronic non-coding RNA (Cui et al., 2017). The functional roles of lncRNAs in plants are increasingly being unraveled, and a number of various reports associated with identification of lncRNAs in multiple plant species (Wang and Chekanova, 2017), including *Zea mays* (Li et al., 2014; Kim et al., 2019), *Solanum lycopersicum* (Cui et al., 2020), *Arachis hypogaea* (Zhao et al., 2019; Tian et al., 2020), *Oryza sativa* (Li et al., 2020), *Populus* (Ma et al., 2019), and *Arabidopsis thaliana* (Zhao et al., 2018), have been published. However, most experimental proofs focus on the functions of lncRNAs associated with plant development and abiotic stress responses, such as fruit ripening (Li et al., 2018; Yu et al., 2019), growth (Meng et al., 2018; Wang Y. et al., 2018), nutritional stimulation (Wang et al., 2017), and salt and drought stresses (Qin et al., 2017). In contrast, there are just beginning to emerge reports about lncRNAs associated with resistance regulatory mechanisms against pathogens (Xin et al., 2011; Zhu et al., 2014). To date, numerous pathogen-responsive lncRNAs have been identified from *Triticum aestivum* infected with powdery mildew and stripe rust (Zhang et al., 2016); *Brassica napus* with *Sclerotinia sclerotiorum* (Jain et al., 2017); *Solanum lycopersicum* with TYLCV (Joshi et al., 2016); *Gossypium* spp. with *Verticillium dahliae* (Wang J. et al., 2018); *Oryza sativa* with *Magnaporthe* (Zhang et al., 2018); and *Oryzae*, *Arabidopsis thaliana* with *Fusarium oxysporum* (Zhu et al., 2014). Kwenda et al. (2016) genome-wide identified potato lincRNAs and functionally analyzed the mechanism in response to *Pectobacterium carotovorum* subspecies *brasiliense* infection. A total of 1342 mRNAs and 22 lncRNAs of rice were identified to be differentially expressed after RBSDV infection (Zhang et al., 2020). An lncRNA of rice, ALEX1, whose expression is highly induced by Xoo infection, can active the JA pathway and promote resistance to bacterial blight (Yu et al., 2020). The differentially expressed lncRNAs (DELs) were identified, and lncRNA-mRNA networks were examined by performing comparative transcriptome analysis of *P. infestans*-resistant and -susceptible tomatoes. The tomato lncRNA16397 was confirmed to reduce reactive oxygen species accumulation and alleviate cell membrane injury by inducing SlGRX expression, leading to enhanced resistance to *P. infestans* (Cui et al., 2017). During *P. infestans* infection, the tomato Sl-lncRNA15492 inhibits the homeostasis of Sl-NBS-LRR1 through the infection of Sl-miR482a (Jiang et al., 2020). In the tomato, lncRNA33732 activated by transcription factor WRKY1 can induce H_2_O_2_ accumulation and participate in the mechanism of resistance to *P. infestans* (Cui et al., 2019). Cui et al. (2020) found that lncRNAs might regulate ceRNAs to decoy microRNAs (miRNAs) and function as their target genes in tomato plants, increasing resistance to *P. infestans*. In addition to mRNA and ceRNAs, other types of RNA were regulated by lncRNA to participate in the plant response to pathogen infection, such as circRNAs, and sRNAs (Zhou et al., 2020).

Although several studies have confirmed that lncRNAs play an important role in multiple plants’ resistance to pathogen infection, the regulatory mechanism of lncRNAs remains poorly understood. To date, compared with animal species, genome-wide identification and characterization of lncRNA in plants is still in its infancy (Kim and Sung, 2012). In this work, we genome-wide identified potato lncRNAs and investigated the regulatory mechanism of potato response to *P. infestans* infection for the first time. Our results provide insights into the valuable information for the basal plant defense mechanisms of lncRNAs and can benefit future molecular-based breeding approaches to acquire pathogen-resistant plants.

## Materials and Methods

### Transcriptome Data Collection

The Sequence Read Archive of the National Center for Biotechnology Information (NCBI) collection was used to obtain RNA data sets of potato (PRJNA203403), which were used to identify transcripts of the potato in response to *P. infestans* infection. WT (Russet Burbank) is the tuber late blight–susceptible non-transformed line, and RB tissue is the tuber late blight–resistant transgenic Russet Burbank line. Potato tubers of RB tissues infected by *P. infestans* or mock for 0, 24, and 48 h were named ht0/mht0, ht3/mht3, and ht4/mht4, respectively. Potato tubers of WT infected by *P. infestans* or mock for 0, 24, and 48 h were named rt0/mrt0, rt3/mrt3, and rt4/mrt4, respectively (Supplementary Table 1; Gao et al., 2013). There were three biological replicates for each time point.

### LncRNA Identification and Target Gene Prediction

We examined the quality of all RNA sequences by performing FASTQC software (v. 0.11.9)^1^. Trimmomatic software (v. 0.39) was used to remove the adaptors and low-quality bases (Bolger et al., 2014), and the command was trimmomatic-0.39.jar PE-threads 5 sample1.R1.fq sample2.R2.fq sample1.R1.clean.fq sample1.R1.unpaired.fq sample1.R2.clean.fq sample1.R2. unpaired.fq AVGQUAL:30 1> sample1.QC.log 2 > &1. The unpaired reads were discarded. We used the spliced read aligner HISAT (v2.1.0) (Pertea et al., 2016) to align clean reads from all samples to the potato reference genome (v4.03)^2^ released in 2011 (Xu et al., 2011) (hisat2 -p 10 –dta –x database –q -1 sample1.R1.clean.fq -2 sample1.R2.clean.fq -S sample1.sam –novel-splicesite-outfile sample1.splicesite 2> align_summary.txt). StringTie software (v2.1.3) was used to assemble the transcripts of each experiment separately (Pertea et al., 2016) (stringtie sample1.sorted.bam -p 10 -G potato_GM_V403.gtf -o sample1.transcript.gtf -l sample1). The assembled transcript isoforms detected in only one sample were removed to reduce transcriptional aberration. The assembled transcript isoforms were compared with the potato genome annotation by using Gffcompare (v. 0.11.2) (gffcompare -r potato_GM_V403.gtf -o./merged stringtie_merged.gtf). The assembled transcript sequences were uploaded to Transcriptome Shotgun Assembly (TSA) of NCBI (GIXO00000000).

All transcripts from the transcriptome assemblies were used to identify lncRNAs using FEELnc software (v. 0.1.1). In the first step, the short (<200 bp) and single-exon transcripts were filtered using FEELncfilter (Harrow et al., 2012). In the second step, after the transcriptome reconstruction, we used FEELnccodpot predictors to compute a coding potential score of the assembled sequences. The UniProt database and CPC2 server were used to calculate the coding potential and remove transcripts with open reading frame (ORF) greater than 100 amino acids. We used blastn to filter the transcripts overlapping all mature miRNA sequences from miRBase, a high-confidence subset of miRBase obtained from miRBase (Kozomara et al., 2019)^3^ (*E*-value < 1 × 10^–5^). Finally, RNAs with length ≥200 bp, potential coding scores ≤0.5, coding potential <0, ORF <100 amino acids, transcripts per million (TPM) > 1 at least one sample, and ORF cover ≤50% are defined as lncRNAs. The target genes of lncRNAs were also predicted. The coding genes within 100 kb 5’ upstream or 3’ downstream of each lncRNA are identified as potential *cis*-targets (Hou et al., 2017). The Pearson correlation between the lncRNA and the gene were calculated based on expression patterns. A high Pearson correlation between lnRNA and gene represents that the expression patterns are similar in all samples, and they are more inclined to play the same role or have a regulatory relationship. Therefore, using highly correlated genes as targets of lncRNA is a good strategy for predicting the function of lncRNA. Genes with a Pearson correlation of *r* > 0.98, *P* ≤ 0.05 to lncRNA were considered as potential *trans* targets for the lncRNA. To further understand the biological process of target genes, we performed Kyoto Encyclopedia of Genes and Genomes (KEGG) and Gene Ontology (GO) enrichment analysis by using KOBAS software 3.0 (prepareKEGGenrich.pl all.genes.list sig.genes.list geneName2KO KO2ko) (Mao et al., 2005; Xie et al., 2011) and topGO software (v 2.42.0) (topGO.enrichment.R -f sig.genes.go -b all.genes.go) (Frazee et al., 2015), respectively.

### Identification of Alternative Splicing Events From All LncRNAs in Potato

We used the ASTALAVISTA program^4^ to identify alternative splicing (AS) events (astalavista -t asta -i stringtie_merged.gtf -d 0 -o as.gtf.gz). The diverse categories of AS events, alternative acceptor (AA), alternative donor (AD), intron retention (IR), exon skipping (ES), and mutually exclusive exons (MX) (Feng et al., 2019) were identified using a Perl script generated in house.

### Differential Abundance of LncRNAs

We used Salmon (v 0.14.1) software to quantify all assembled transcripts, and the TPM was used to express the abundance (salmon quant -i transcripts_index -l A -1 reads_1.fastq -2 reads_2.fastq -o transcripts_quant) (Patro et al., 2017). We used tximport (v 1.18.0) to import transcript-level abundance and summarize it into expression matrices for downstream analysis. The read count of lncRNAs was used to calculate the difference by performing the R package DEseq2 (v1.30.0) with *P*-value ≤ 0.05 and log_2_ (fold change) > 1 (DEseq2.R rcound.txt group) (Love et al., 2014). R packages circlize(), ggplot(), and TCseq were used to plot distributions of lncRNAs and mRNAs in all potato chromosomes, bubble charts, heat maps, and expression pattern maps, respectively. A Venn gram was plotted by using TBtools software (v1.051) (Chen et al., 2020). We used Cytoscape software (v3.7.2) to construct an interactive network between lncRNAs and pathways associated with targets (Shannon et al., 2003). Gene regulatory networks (GRN) were generated by performing R package GENIE3 (v1.12.0) (Huynh-Thu and Geurts, 2018). Briefly, the transcripts with TPM > 1, at least one sample, and 16 lncRNAs from cluster 2 were selected to construct GRN based on expression patterns. We used GENIE3 to estimate the random forest regression for each transcript based on the lncRNAs as inputs and the default parameters (K = sqrt, nb.trees = 1000, input.idx = list of lncRNAs, importance. measure = IncNodePurity, seed = NULL) were applied^5^. Subsequently, the connectivity between transcript and lncRNA was calculated by GENIE3 software. For each lncRNA, all predicted targets (connectivity >0.3) were extracted (Ramírez-González et al., 2018).

### Plant Materials

The potatoes Désirée/Eshu 3 were maintained at the Shandong Agricultural University, Tai’an City, China, as the research material in this study. We propagated potato plantlets on Murashige-Skoog medium under growth chambers with 16 h of light at 20°C. Three-week-old plantlets were transplanted in a greenhouse and grown in individual pots at 20–26°C for 4–5 weeks for further assays. *Nicotiana benthamiana* plants were used to perform transient overexpression assays and grown in a long-day glass house with 16 h of light at 22°C; light intensity and humidity were 145 mE m^–2^ s^–1^ and 40%, respectively. The 5-week-old *N. benthamiana* was selected for *P. infestans* colonization and *Agrobacterium tumefaciens* infiltration.

### Vector Construction and *Agrobacterium tumefaciens* Transformation

The primers of *StLNC0004* were designed (forward: acgggg gactctagaggatccAGAAATTGTGCAACTTCTATAGACATATCA, reverse: cgatcggggaaattcgagctcTCTCCTCCACCACACCATCA TC) and used to amplify the full length of *StLNC0004* by performing PCR, and the products were recombined into the expressed vector pROK II to generate pROK II:StLNC0004 using the ClonExpress^®^ Entry One Step Cloning Kit (Vazyme^®^, Vazyme Biotech Co., Ltd., China). We sequenced the constructed transient overexpression vectors for further confirmation. The electroporation was performed to transform pROK II:StLNC0004/pROK II into *A. tumefaciens* strain GV3101.

### *Agrobacterium*-Mediated Transient Expression

We carried out the *A. tumefaciens* transformation and *P. infestans* infection assays on *N. benthamiana* as described previously (Bos et al., 2010; Saunders et al., 2012; McLellan et al., 2013). Briefly, we used yeast extract and beef medium with appropriate antibiotics to culture the *A. tumefaciens* strains containing recombined vectors at 28°C (200 rpm, 24 h). We used sterile 10 mM MES and 10 mM MgCl_2_ buffer containing 200 μM acetosyringone to resuspend the bacteria pellet. Bacterial optical densities of OD_600_ = 0.3 were used for transient expression and *P. infestans* pathogenicity assays in *N. benthamiana* (Zhong et al., 2018).

### *Phytophthora infestans* Production

The *P. infestans* isolate HLJ, a strongly pathogenic oomycete, was conserved at the State Key Laboratory of Shandong Agricultural University, Tai’an, Shandong, China (Wang et al., 2019). *P. infestans* HLJ was grown in Petri dishes with Rye A for 2 weeks at 18°C. Then, we used 5 mL sterile water to flood the dishes and used a glass rod to scrape sporangia for release. The clean Petri dish was poured into suspension, placed on ice, and stored at 4°C for 3 h to release the zoospores. We collected the resulting solution in a falcon tube and counted sporangia numbers by using a hemocytometer and adjusted to 15,000 sporangia/ml.

### Identification of Susceptible/Resistant Materials

We placed the potato leaves in a Petri dish covered with moist filter paper and placed 3 leaves of the same cultivar in each dish. When inoculating, we dropped 10 μl of *P. infestans* HLJ spore suspension on both sides of the main vein of the leaves. After inoculation, we cultured at 18°C in a light incubator and inspected the disease condition of leaves after 5–7 days to determine whether it was pathogenic. Potatoes with diseased leaves and sporangia are susceptible materials, and leaves with only necrosis and no sporangia are resistant materials.

### *Phytophthora infestans* Inoculation

The potato leaves were inoculated with *P. infestans* at a concentration of 4 × 10^5^ sporangia ml^–1^ for real-time quantitative PCR. Reverse transcription-polymerase chain reaction (RT-PCR) was used for the colonization of *P. infestans* in potatoes, and the primers of the conserved regions of the PI-O8 gene (Wang et al., 2019) of the *P. infestans* strains were designed (F: AAGATGATGTTGGATGATTG, R: TGCCTGATTTCTACCTTCT, 250 bp). Tobacco leaves were removed 24 h after infection and placed in sealed boxes. Each infiltration site was inoculated with 10 μl zoospores from *P. infestans* isolate 4 × 10^4^ sporangia/ml (Zhong et al., 2018). As previously described, the degree of disease development was recorded in infected leaf assays (He et al., 2015; Tian et al., 2015). Fifty leaves from 15 individual plants were used for each of three replicates. ANOVA was used to analyze all data.

### RNA Extraction

According to the manufacturer’s instructions on *AG RNAex Pro* Reagent AG21101 [Accurate Biotechnology (Hunan) Co., Ltd], we extracted the total RNA from all potato and *N. benthamiana* samples. We used the Agilent 2100 Bioanalyzer (Agilent Technologies, Santa Clara, CA, United States) to analyze the integrity, quality, and concentration of the RNAs.

### Real-Time Quantitative PCR Analysis

The Evo M-MLV RT Kit with gDNA Clean for qPCR AG11705 [Accurate Biotechnology (Hunan) Co., Ltd] was used to reverse-transcribe the total RNA into a single-stranded RNA. We used Primer-BLAST in NCBI^6^ to design the gene-specific primers. Elongation factor 1-alpha-like protein (EF1α) was used as an internal reference gene. The reactions were conducted in a 20 μL volume containing 10 μL SYBR Green PCR Master Mix, 1 μL of each primer (10 μmol/ml), 3 μL dd H_2_O, and 5 μL of the template cDNA under the following conditions: 30 s at 72°C, 35 cycles of 5 s at 95°C, 30 s at 55°C, and 30 s at 72°C. Real-time RT-PCR was conducted using LightCycler96 (Switzerland). The real-time PCR analysis was performed using three biological replicates for each treated sample and at least three technical replicates of each biological replicate. Relative expression levels of the target genes were calculated using the 2^–ΔΔCt^ method (Livak and Schmittgen, 2001).

### Statistical Analysis

The results are expressed as mean ± standard deviation (SD). The one-way ANOVA and Tukey’s test were used to analyze the differences between groups. *P*-value ≤ 0.05 is considered to be statistically significant.

## Results

### Genome-Wide Identification of LncRNAs in Potato

**FIGURE 1:**
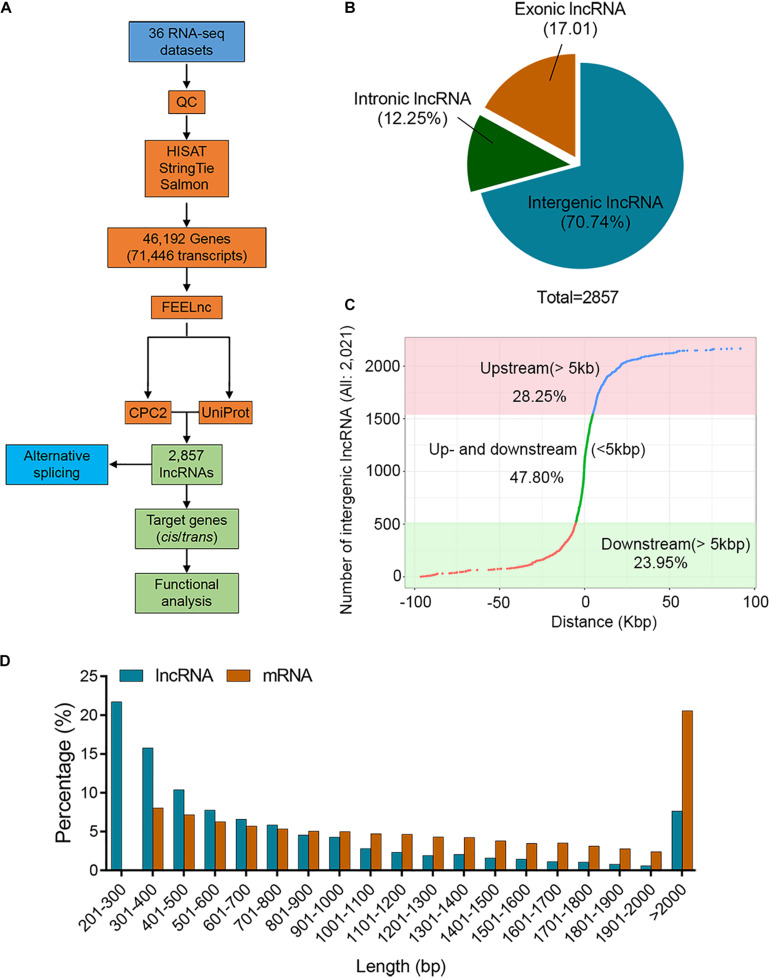
Identification of potato lncRNAs. **(A)** Pipeline for the identification of lncRNAs in the potato. **(B)** Proportion of lncRNAs located within 5 kb (upstream or downstream) or further than 5 kb from the nearest protein-coding genes. **(C)** Distribution of lincRNAs located upstream or downstream >5 kb and <5 kb. The *X*-axis represents the distance between the lncRNA and the nearest coding gene on the chromosome. The positive number represents that the lncRNA is located upstream of the gene, and a negative number represents downstream of the gene. The lncRNA with zero distance from the nearest gene belongs to intronic or exonic lncRNA. **(D)** Histogram showing the distribution of lncRNA and mRNA lengths.

To achieve a relatively comprehensive set of potato lncRNAs, we collected 36 public Illumina transcriptomes (Supplementary Table 1) and used the HISAT-StingTie-Salmon-FEELnc-CPC-Uniprot pipeline for further analysis (Figure 1A). After quality control, 0.85 billion clean reads (average 23.59 million) were obtained for lncRNA identification. The “Q30” value ranged from 97.72 to 98.32%, and their GC content ranged from 42.08 to 44% (Supplementary Table 2). All clean reads were mapped to the potato reference genome using HISAT2 software, and on average, 84.10% of the reads were successfully aligned with the potato reference genome sequence (Supplementary Table 3). Subsequently, we performed the assembly process using Stringtie software, and a total of 46,192 genes (71,446 transcripts) containing 33,150 mRNA were generated (Figure 1A and Supplementary Table 4). The transcript with TPM value >1 for at least one sample was considered to be expressed. The selected transcripts associated with each sample suggest that the distribution of transcript abundance in all samples was broadly similar (Supplementary Figure 1 and Supplementary Table 5). In total, there were 2857 lncRNAs (length ≥200 bp, ORF cover ≤0.5, potential coding scores ≤0.5, TPM > 1, coding potential <0) generated by using FEELnc, CPC2 software, and the UniProt database, and all lncRNAs were renamed (Supplementary Table 6). The lncRNAs were characterized according to the locations relative to the nearest protein-coding genes. The majority of lncRNAs (70.74%) were located in intergenic regions, and 29.26% of the lncRNAs (exonic: 17.01%, intronic: 12.25%) overlapped with protein-coding genes (Figure 1B). In all, 2021 intergenic lncRNAs, 28.25% and 23.95%, were at least 5 kb away upstream and downstream of genes, respectively. The remaining 47.80% of intergenic lncRNAs were located within 5 kb of genes (Figure 1C). The lengths of 76.79% lncRNAs ranged from 200 to 1000 bp; however, 57.43% mRNAs were longer ranging than 1000 bp (Figure 1D), suggesting that the sequence components of the mRNA exhibit differently when compared with the lncRNAs.

### Characterization of Potato LncRNAs

**FIGURE 2:**
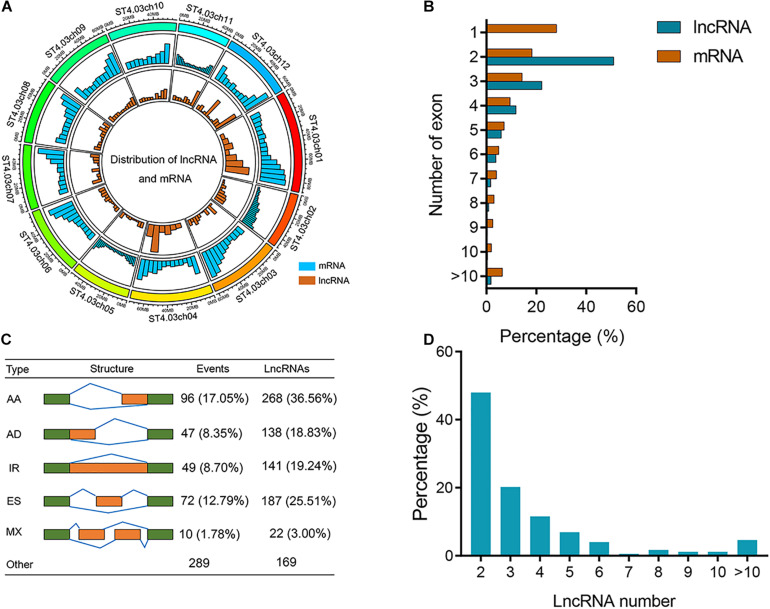
Characterization of potato lncRNAs. **(A)** Distribution of lncRNAs and mRNA along each potato chromosome. The abundance of lncRNA and mRNA in physical bins of 10 Mb for each chromosome (generated using Circlize). **(B)** Percentage of lncRNAs and mRNAs containing different numbers of exons. **(C)** Classification of AS events. Cartoons show five major types of AS events, including IR, AD, AA, ES, and MX. The numbers of AS events and associated lncRNAs are shown. Numbers in parentheses show the proportions of lncRNAs generated from different types of AS events occupying all AS lncRNAs. **(D)** Percentage of genes containing different numbers of lncRNAs.

We used R package Circlize (v. 0.4.10) to measure the distributions of lncRNA and mRNA on potato chromosome (Figure 2A). Higher densities of mRNA and lncRNAs were observed in the chromosome “arms” of most potato chromosomes than pericentromeric regions (Figure 2A). A total of 9323 (28.12%) mRNAs were composed of a single exon, and approximately 51.00% of lncRNAs were composed of two exons (Figure 2B). We detected 2491 lncRNAs derived from 563 AS events associated with 173 genes (Supplementary Table 7). Subsequently, the five major AS events, AA, AD, IR, ES, and MX, were identified by customizing a user-friendly program (Figure 2C). Figure 2C shows the statistics of AS events and corresponding gene models in the potato. We found that 49 (8.70%) IR events were identified with one retained intron for each transcript. There were 1158 (46.49%) lncRNAs undergoing AS associated with AA events (Figure 2C). The number of lncRNAs generated from ES was the second most common of AS events. The percentage of genes with two lncRNAs was significantly higher than other genes (47.98%) (Figure 2D).

### Predicting the Target Genes of the LncRNAs

**FIGURE 3:**
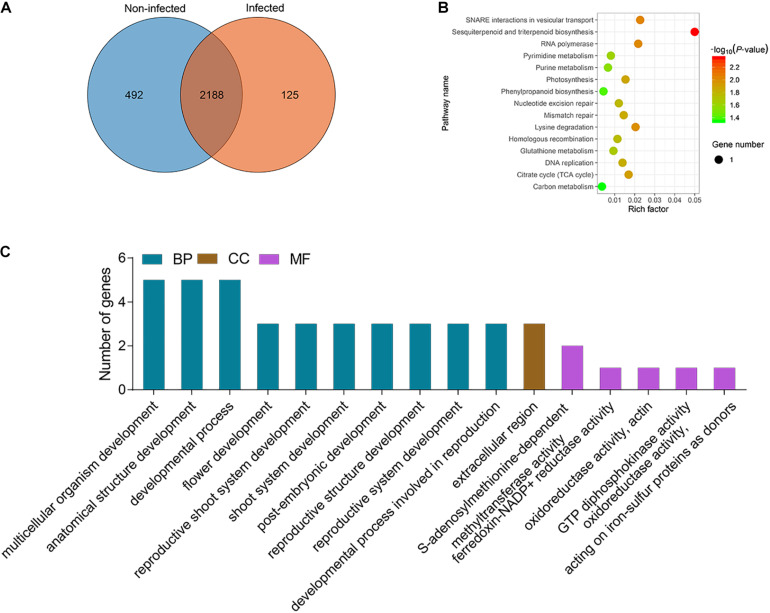
Analysis of specific lncRNAs from *P. infestans* infected samples. **(A)** Venn gram showing the distribution of lncRNAs in infected (ht3, ht4, rt3, and rt4) and non-infected (ht0, rt0, mht0, mht3, mht4, mrt0, mrt3, and mrt4) samples. **(B)** KEGG enrichment analysis of targets of 213 specific lncRNAs from infected samples. The circle size represents the gene number. Green to red represents the low to high *P*-value. **(C)** GO terms enrichment analysis of targets of 125 specific DELs and the top 10 of biological process (BP), and top 5 of molecular function (MF) and cellular component (CC) are shown.

There was no information about functionally characterized lncRNAs in potato. Therefore, to investigate the potential functional roles or biological processes of the lncRNAs, their target genes were identified as *cis* and *trans* (Supplementary Table 8). LncRNAs with TPM ≥ 1 in at least one sample were considered to be expressed, and all samples were divided into two categories: infected samples, including ht3, ht4, rt3, and rt4, and non-infected samples, including ht0, mt0, mt3, mt4, rt0, mrt0, mrt3, and mrt4. The lncRNAs with TPM > 1 in at least one sample of infected samples and TPM ≤ 1 in all samples of non-infected samples were defined to be specifically expressed lncRNAs in infected samples. We analyzed the distribution of lncRNAs in two types of potato tissues and found that a total of 125 lncRNAs were specifically expressed in infected samples (Figure 3A). To understand the function of the specific lncRNA, we performed KEGG pathway and GO term enrichment analysis of the targets of these specific lncRNAs (Supplementary Table 9). The KEGG pathway analysis showed that a total of 15 pathways were enriched significantly (*P*-value ≤ 0.05), including “Sesquiterpenoid and triterpenoid biosynthesis,” “SNARE interactions in vesicular transport,” and “RNA polymerase” (Figure 3B). A total of 11, 1, and 32 GO terms for molecular function (MF), cellular component (CC), and biological process (BP) were obtained, respectively (Supplementary Table 9). For biological process, major categories were found for multicellular organism development (GO:0007275), anatomical structure development (GO:0048856), and developmental process (GO:0032502). For the cellular component, genes were only involved in the extracellular region (GO:0005576). For molecular function, S-adenosylmethionine–dependent methyltransferase activity (GO:0008757) was the most represented GO term, followed by ferredoxin-NADP+ reductase activity (GO:0004324) (Figure 3C).

### Analysis of DELs

**FIGURE 4:**
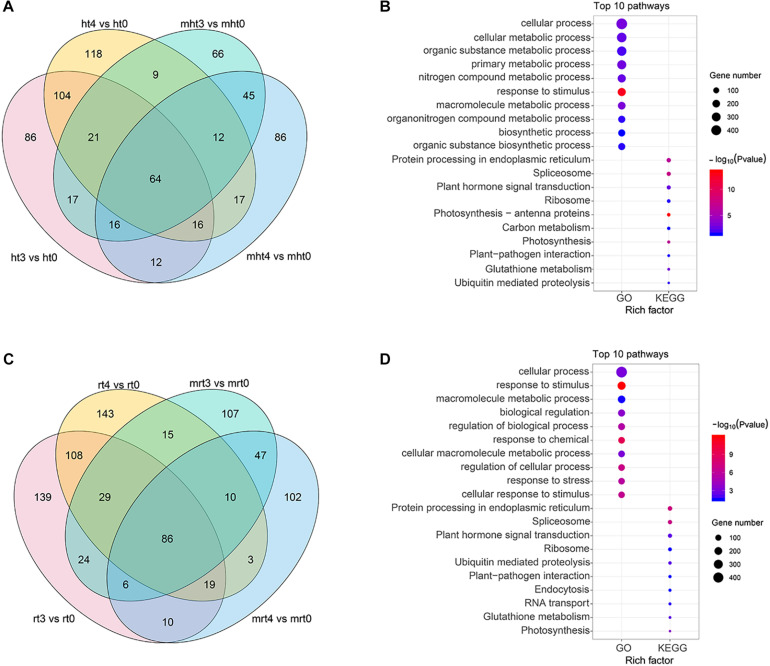
Identification and characterization of DELs. **(A,C)** Venn gram showing the distribution of DELs in WT and RB tissues, respectively. **(B,D)** GO and KEGG enrichment analysis were performed, and the top 10 GO terms and KEGG pathways are shown. The circle size represents the gene number. Blue to red represents the low to high *P*-value.

To identify DELs between 24 h (ht3, rt3, mht3, and mrt3) and 48 h (ht4, rt4, mht4, and mrt4) samples and 0 h (ht0, rt0, mht0, and mrt0) samples, lncRNAs with at least a 2.0-fold change in expression and *P*-value ≤ 0.05 were considered to be differentially expressed (Supplementary Figure 2 and Supplementary Table 10). We analyzed the distribution of DELs in RB tissue and WT, respectively. In total, 308 specific DELs in RB tissue infected with *P. infestans*, including 86 specific DELs from ht3 vs. ht0, 118 specific DELs from ht4 vs. ht0, and 104 share DELs from the two comparison groups, were obtained (Figure 4A). These specific DELs may be related to the potato response to *P. infestans* infection. Furthermore, we investigated the functional roles of the specific DEL targets by KEGG and GO enrichment analysis (Supplementary Table 11). We found that multiple KEGG pathways and GO terms were significantly enriched, such as KEGG pathways “Photosynthesis-antenna proteins” and “Spliceosome” and GO terms photosynthesis and protein-chromophore linkage (Figure 4B). We observed that a total of 390 specific DELs (139 DELs in rt3 vs. rt0, 143 DELs in rt4 vs. rt0, both: 108 DELs in both comparisons) may be involved in the potato response to *P. infestans* infection in WT samples (Figure 4C). Functional enrichment analysis shows that KEGG pathways “Protein processing in endoplasmic reticulum” and “Spliceosome” and GO terms cellular process and response to stimulus were enriched significantly (Figure 4D and Supplementary Table 12).

### Characterization of Specific DELs in Resistance Sample

**FIGURE 5:**
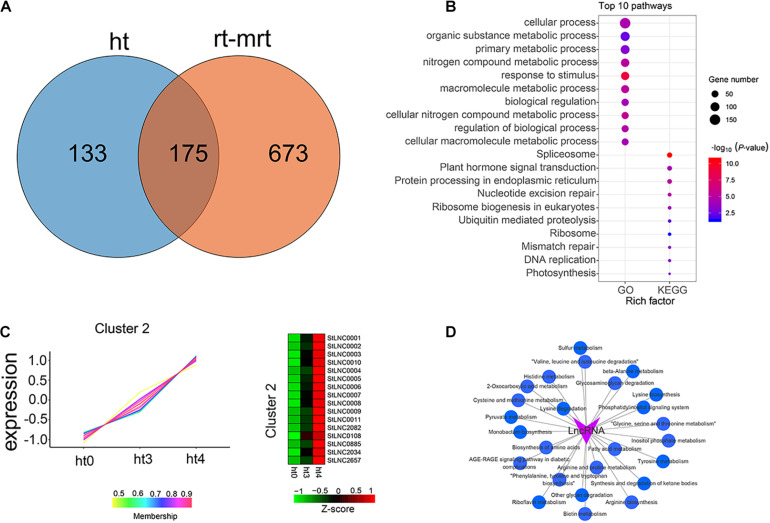
Identification and characterization of specific lncRNAs from *P. infestans*–infected RB tissue. **(A)** Venn gram showing the distribution of DELs in ht (*P. infestans*-infected RB tissues) and rt-mrt (*P. infestans*-infected WT and mock-treated RB tissues and WT). **(B)** GO and KEGG enrichment analysis of targets of 133 specific DELs in RB tissue infected by *P. infestans* and the top 10 pathways are shown. The circle size represented the gene number. Blue to red represents the low to high *P*-value. **(C)** TCseq was performed to cluster 133 specific DELs, and heat maps show the expression patterns of lncRNAs from cluster 2. Green color represents the low expression level and red is the high expression level. **(D)** The interactive network between lncRNAs from cluster 2 and the significantly enriched pathways.

To identify the potential functional roles of specific DELs in the resistant sample of RB tissue, we analyzed the distribution of specific DELs in RB tissue infected by *P. infestans*. Figure 5A shows that a total of 133 DELs were observed only in RB tissue, indicating these DELs may participate in the resistance mechanism of the potato to *P. infestans* infection. Many KEGG pathways and BP of GO terms were involved in the targets of these specific DELs, such as “Plant hormone signal transduction,” “Ubiquitin mediated proteolysis,” and “Photosynthesis” from KEGG pathways and response to stimulus and biological regulation from GO terms (Figure 5B). The expression patterns of the specific DELs were clustered into six types by performing R package TCseq (Supplementary Figure 3). The expressed levels of 16 DELs in cluster 2 were increased at 48 h compared with 0 h (Figure 5C). To further explore the regulatory patterns of lncRNAs from clusters 2 (regulatory factors) and mRNA (target genes) during *P. infestans* infection in the potato, we used GENIE3 to generate GRNs, which are directed networks of lncRNAs and their regulatory genes (Supplementary Table 13). As the top-performing method in the DREAM4 and -5 GRN reconstruction challenges, GENIE3 takes advantage of the random forest machine learning algorithm. All targets were subjected to KEGG enrichment analysis and the interactive network between lncRNAs, and a total of 25 significantly enriched pathways were constructed by Cytoscape 3.7.2 software (Figure 5D). The lncRNAs may participate in the potato resistance mechanism by regulating these pathways.

### Verification of DELs by qRT-PCR

**FIGURE 6:**
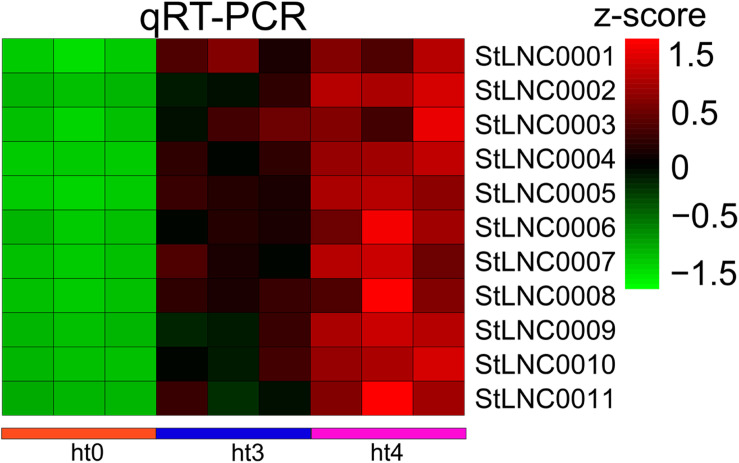
Comparison of 11 specific DELs from cluster 2 expressions determined by RNA-seq and qRT-PCR. Relative transcript levels of the DELs were determined by qRT-PCR. Data is shown by a heat map with *z*-score.

The expression patterns of 11 specific DELs from cluster 2 were randomly selected to identify by performing qRT-PCR, and the lncRNAs were renamed (Supplementary Table 14). EF1α was used as the reference gene. Because the potato materials (WT/RB) used in this work were not obtained, we used leaves of susceptible/resistant potatoes Désirée/Eshu 3 identified by detecting separated leaves (Supplementary Figure 4) to detect the expressed levels of selected lncRNAs. Sequence comparison showed that the full length of the 11 lncRNAs in the RNA-seq data and PCR results from Désirée and Eshu 3 share high nucleic acid similarity (99%) (Supplementary Table 13). The RT-PCR data indicates the colonization of *P. infestans* in potato leaves was successful (Supplementary Figure 5). The selected lncRNAs were upregulated in resistant potatoes infected by *P. infestans* (Figure 6), and there was no significant difference in the susceptible potato, indicating that the DELs obtained in this study can be used for research on the Désirée/Eshu 3 potatoes.

### Transient Expression of lncRNAs Enhances the Resistance of *N. benthamiana* to *P. infestans*

**FIGURE 7:**
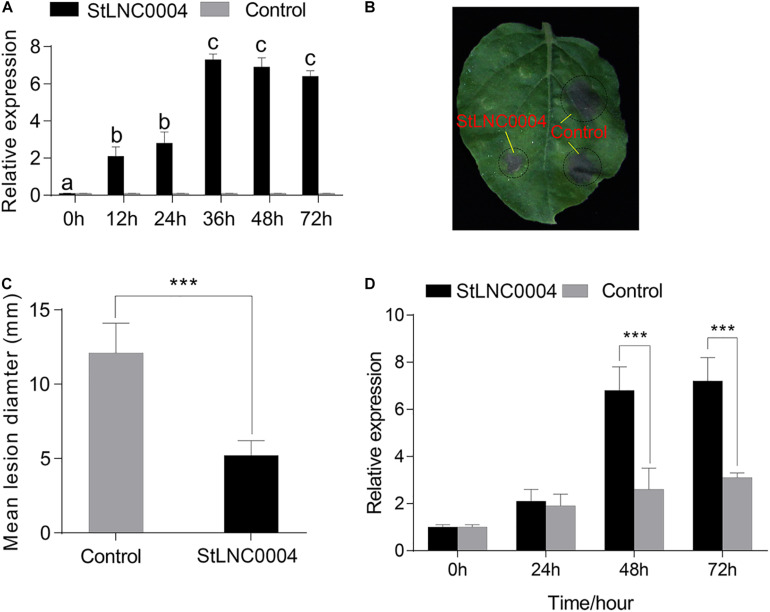
Transient expression of *StLNC0004* inhibits *P. infestans* colonization in *Nicotiana benthamiana*. **(A)** Quantitative real-time PCR (qRT-PCR) was performed to identify the expression level of *StLNC0004* in *N. benthamiana*. StLNC0004 and Control represent the *N. benthamiana* containing pROK II: StLNC0004 and pROK II, respectively. **(B)** Representative leaf images of *P. infestans* lesions following expression of StLNC0004/Control in *N. benthamiana*. **(C)** Mean *P. infestans* lesion diameter (mm) on sites of transient expression of the StLNC0004 and Control. **(D)** QRT-PCR identified the expression level of *NtEXT*, the ortholog of *StEXT*, the target gene of *StLNC0004*. Different letters in the columns represent a significant difference in the qRT-PCR results between StLNC0004 and Control (*P* ≤ 0.05). *** represents a significant difference between StLNC0004 and Control (*P* ≤ 0.001).

The expression level of *StLNC0004* at 24 and 48 h from RNA-seq and qRT-PCR data was highest among the 11 specific DELs from cluster 2; therefore, we selected *StLNC0004* to further investigate the potential function in response to *P. infestans* in *N. benthamiana*. The transcription level of *StLNC0004* was significantly increased after *A. tumefaciens* strain GV3101 containing pROK II:StLNC0004 transformed into *N. benthamiana* for 36 h (Figure 7A). Then, we carried out the *P. infestans* pathogenicity assays and found that transient overexpression *StLNC0004* in *N. benthamiana* significantly suppresses *P. infestans* growth compared with empty vector (pROK II) control (Figures 7B,C). The result suggests that *StLNC0004* act as positive regulators in *N. benthamiana* resistance to *P. infestans*. The predicted target of *StLNC0004* was gene PGSC0003DMG400000776 encoding extensin precursor (*StEXT*). To obtain the ortholog of *StEXT* from *N. benthamiana*, we blasted the coding region of *StEXT* in the *N. benthamiana* Genome and Transcriptome^7^ and found that the nucleic acid of *StEXT* and *NbEXT* (Nbv5.1tr6234951) were highly similar (80%), indicating that *NbEXT* was the ortholog of *StEEXT*. After infection with *P. infestans* for 48 and 72 h, the expression level of *NbEXT* in *N. benthamiana* containing pROK II:StLNC0004 was significantly higher than that of the control (Figure 7D). This suggests that *StLNC0004* may act as a positive regulator in potato resistance to *P. infestans* by regulating the transcription level of *StEXT*.

## Discussion

In general, the functions of most of the lncRNAs that have been identified are still unstudied. The lncRNAs responsive to *Pectobacterium carotovorum* subspecies *brasiliense* infection in potato and *P. infestans* infection in tomato were identified, but no information on lncRNAs associated with the potato response to *P. infestans* infection is available. In this work, we obtained 36 published RNA-seq data sets from different cultivars of potato treated with *P. infestans* or mock from NCBI. We identified novel candidate lncRNAs that might be important components in potato defense response mechanisms during *P. infestans* infection.

A total of 2857 candidate lncRNAs from all samples were identified in this study. The average length of mRNAs in potato plants was longer than that of lncRNAs, and the percentage of lincRNA was 70.74%. Total lncRNAs distributed throughout 11 potato chromosomes suggest that lncRNA was the functional component of the potato genome. AS is an important functional role of post-transcriptional gene regulation (Reddy et al., 2013). Previous reports prove that AS events can regulate multiple aspects of plants, such as development, growth, flowering, environmental cue responses, circadian clock function, signal transduction, and plant immunity (Remy et al., 2014; Yang et al., 2014; Zhang et al., 2014; Tang et al., 2016). In plants and animals, the transcribed sequences of lncRNAs can be generated by AS events, which are similar to protein-encoding genes (Yang et al., 2017; Ma et al., 2018). LncRNA-PXN-AS1 without exon 4 can inhibit PXN mRNA translation by binding to the coding sequences of the mRNA, and lncRNAPXN-AS1 with exon 4 can protect PXN mRNA degradation, leading to increased expression levels of the mRNA by binding to the 3’ untranslated region of PXN mRNA, preferentially (Yuan et al., 2017). Here, a total of 2491 lncRNAs were generated by 563 AS events from potato RNA-seq data. We found that AA were the most frequently occurring AS events in producing lncRNA. The results suggest that AS events may be an important regulation of lncRNAs.

The functional characterization of potato lncRNAs was improved by predicting target genes in *cis* and *trans*. In total, 308 and 390 specific DELs from RB tissues and WT infected by *P. infestans* at different stages were obtained, respectively, indicating that the specific DELs may respond to *P. infestans* infection in the potato. To further investigate resistance-related lncRNAs, we identified a total of 133 DELs specifically expressed in RB tissue. We used R package TCseq to cluster the DELs and found that the expression of 16 lncRNAs from cluster 2 were higher in ht4 than that in ht0 and ht3, respectively, implying that the 16 lncRNAs may participate in the resistance mechanism in the potato to *P. infestans*. We constructed a directed network of the 16 lncRNAs and their regulated genes by performing GENIE3. A total of 92 KEGG pathways were significantly enriched by the regulated genes of the 16 lncRNAs. The pathway “Plant hormone signal transduction” was enriched with 5 targets, including PGSC0003DMT400004165 encoding SAUR family protein, PGSC0003DMT400064343 encoding the auxin responsive GH3 gene family, PGSC0003DMT400021210 encoding protein phosphatase 2C (PP2C), PGSC0003DMT400049445 encoding DELLA protein, and PGSC0003DMT400058305 encoding the two-component response regulator ARR-A family. The SAUR family may integrate various hormonal and environmental signals to regulate leaf senescence in *Arabidopsis* (Wen et al., 2020). The PP2C-SnRK2-ABF signaling module plays an important role in the ABA signaling pathway (Sun et al., 2020), which can promote plant defense to biotic and drought stresses by regulating stomatal opening and closure (Lim et al., 2015). To investigate the potential regulatory mechanism of lncRNAs, we performed the transient expression assay in *N. benthamiana* using the lncRNA *StLNC0004* with the highest expression level in the 11 lncRNAs from cluster 2 and found that *StLNC0004* inhibited the colonization of *P. infestans* on *N. benthamiana*. The target gene of *StLNC0004* was extensin precursor (*StEXT*), which participates in the plant response to multiple stresses, such as drought (Wu et al., 1996), disease (Ding et al., 2008), and high temperature (Xu et al., 2007). The expression level of *NbEXT*, the ortholog of *StEXT*, was significantly upregulated by *P. infestans* infection in transient expression lines compared with the control, suggesting that *StLNC0004* may enhance the potato defense to *P. infestans* by regulating the transcription level of *StEXT*. These results show that the specifically upregulated lncRNAs may participate in potato resistance mechanisms by regulating many of the targets involved in multiple pathways. The potential lncRNAs of the potato identified from our analysis were relatively robust. These lncRNAs may be used for further functional genomics studies or analysis of potential functional differences between different potato varieties.

## Data Availability Statement

The datasets presented in this study can be found in online repositories. The names of the repository/repositories and accession number(s) can be found below: https://www.ncbi.nlm.nih.gov/, PRJNA203403.

## Author Contributions

CZ and SZ designed the work. LG performed the data analysis. WC and CW wrote the manuscript and performed the experiments. XZ, MZ, and JD edited the manuscript and provided valuable suggestions during the experiment. All authors contributed to the article and approved the submitted version.

## Conflict of Interest

The authors declare that the research was conducted in the absence of any commercial or financial relationships that could be construed as a potential conflict of interest.

## Footnotes

1 http://www.bioinformatics.babraham.ac.uk/projects/fastqc/

2 http://solanaceae.plantbiology.msu.edu/pgsc_download.shtml

3 http://www.mirbase.org/ftp.shtml

4 http://genome.crg.es/astalavista/

5 https://bioconductor.riken.jp/packages/release/bioc/vignettes/GENIE3/inst/doc/GENIE3.html

6 https://www.ncbi.nlm.nih.gov/tools/primer-blast/

7 http://benthgenome.qut.edu.au/

## Supplementary Material

Supplementary Figure 1 The expression level of transcripts in each sample based on log_10_ (TPM).

Supplementary Figure 2 Expression patterns of DELs in each comparison.

Supplementary Figure 3 TCseq wasperformed to cluster specific DELs in RB tissue infected by *P. infestans*.

Supplementary Figure 4 Identifying susceptible/resistant potatoes Désirée/Eshu 3 by detecting separated leaves.

Supplementary Figure 5 Identifying the colonization of *P. infestans* in potato leaves by using RT-PCR.

Supplementary Table 1 Details of each sample.

Supplementary Table 2 Statistics of RNA-seq data.

Supplementary Table 3 Information on Illumina transcriptome-mapped potato reference genome sequence.

Supplementary Table 4 The annotated information on all transcripts.

Supplementary Table 5 The expression level of all transcripts based on TPM.

Supplementary Table 6 Identification of lncRNAs.

Supplementary Table 7 Alternative splicing (AS) event analysis of all lncRNAs.

Supplementary Table 8 Predicting the target genes of lncRNAs.

Supplementary Table 9 KEGG and GO enrichment analysis of specific lncRNAs from *P. infestans*-infected samples.

Supplementary Table 10 Identification of DELs at 24 and 48 h compared with 0 h under the same treatment in WT or RB tissue.

Supplementary Table 11 KEGG and GO enrichment analysis of specific DELs from *P. infestans*–infected samples in RB tissue.

Supplementary Table 12 KEGG and GO enrichment analysis of specific DELs from *P. infestans*–infected samples in WT tissue.

Supplementary Table 13 Constructing GRN using lncRNAs and other mRNAs by performing GENIE3.

Supplementary Table 14 The primers of this work are listed.
