# Supplementary figures and images for: Genome-Wide Identification and Characterization of Potato Long Non-coding RNAs Associated With Phytophthora infestans Resistance
Source: Front Plant Sci. 2021 Feb 10;12:619062. doi: 10.3389/fpls.2021.619062 (PMC7902931; doi:10.3389/fpls.2021.619062)

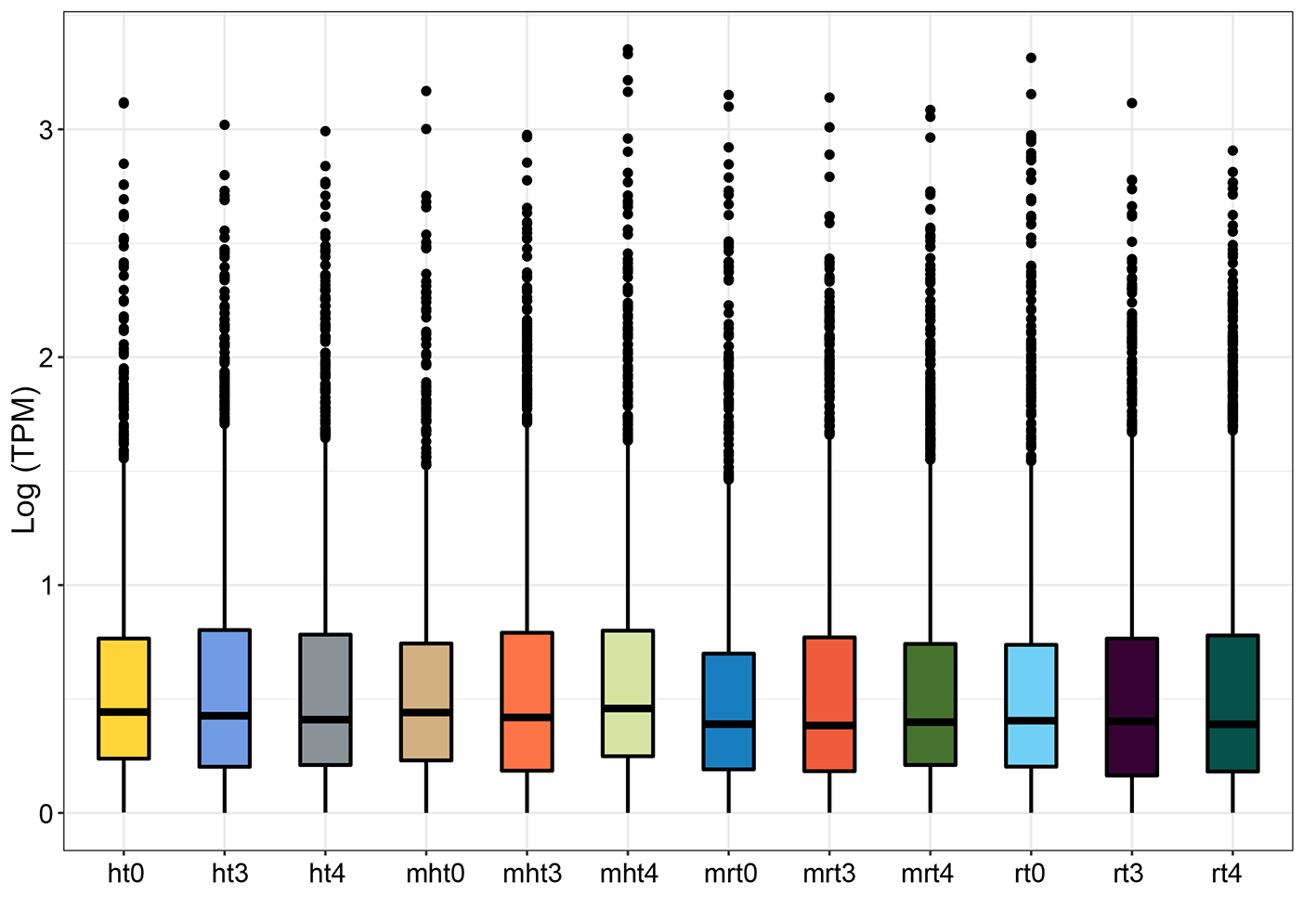

Supplement: Supplementary Figure 1 — The expression level of transcripts in each sample based on log10 (TPM). [file Image_1.TIF]

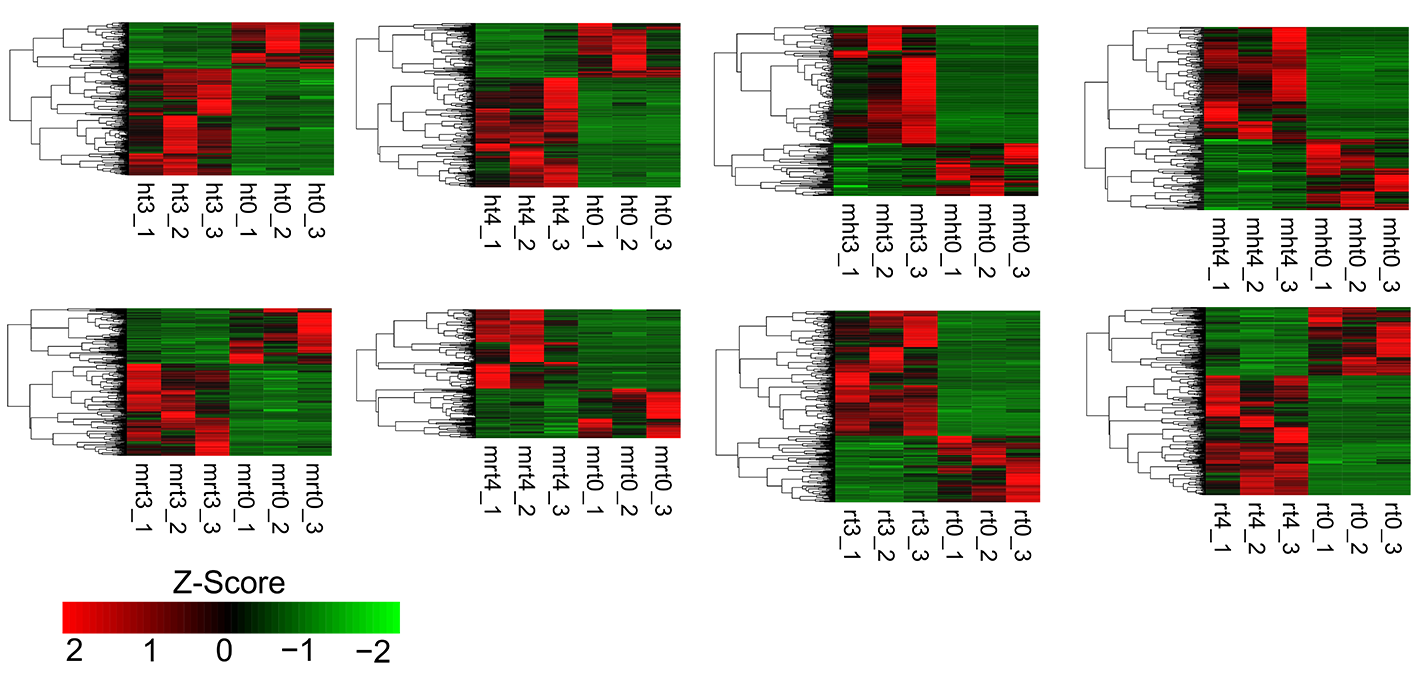

Supplement: Supplementary Figure 2 — Expression patterns of DELs in each comparison. [file Image_2.TIF]

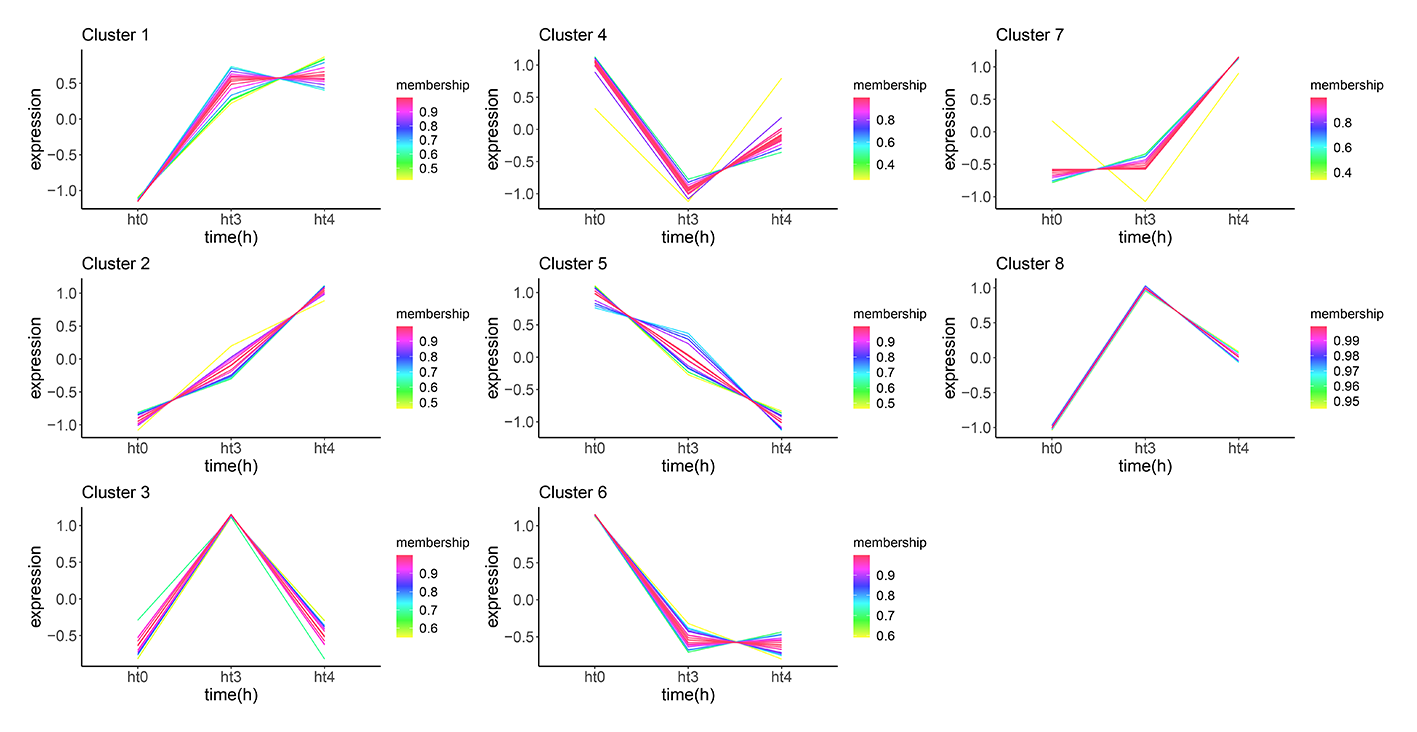

Supplement: Supplementary Figure 3 — TCseq wasperformed to cluster specific DELs in RB tissue infected by P. infestans. [file Image_3.TIF]

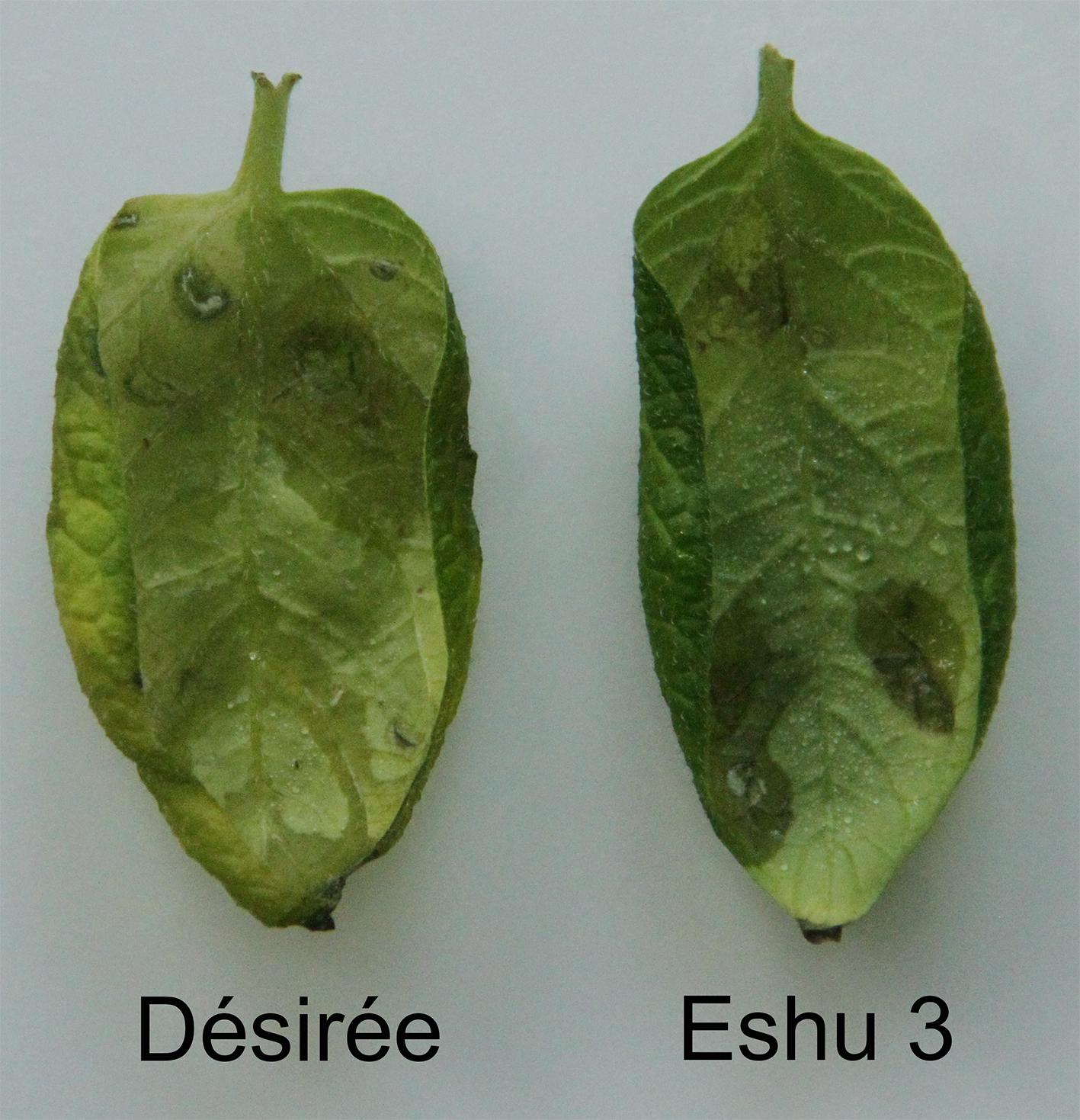

Supplement: Supplementary Figure 4 — Identifying susceptible/resistant potatoes Désirée/Eshu 3 by detecting separated leaves. [file Image_4.TIF]

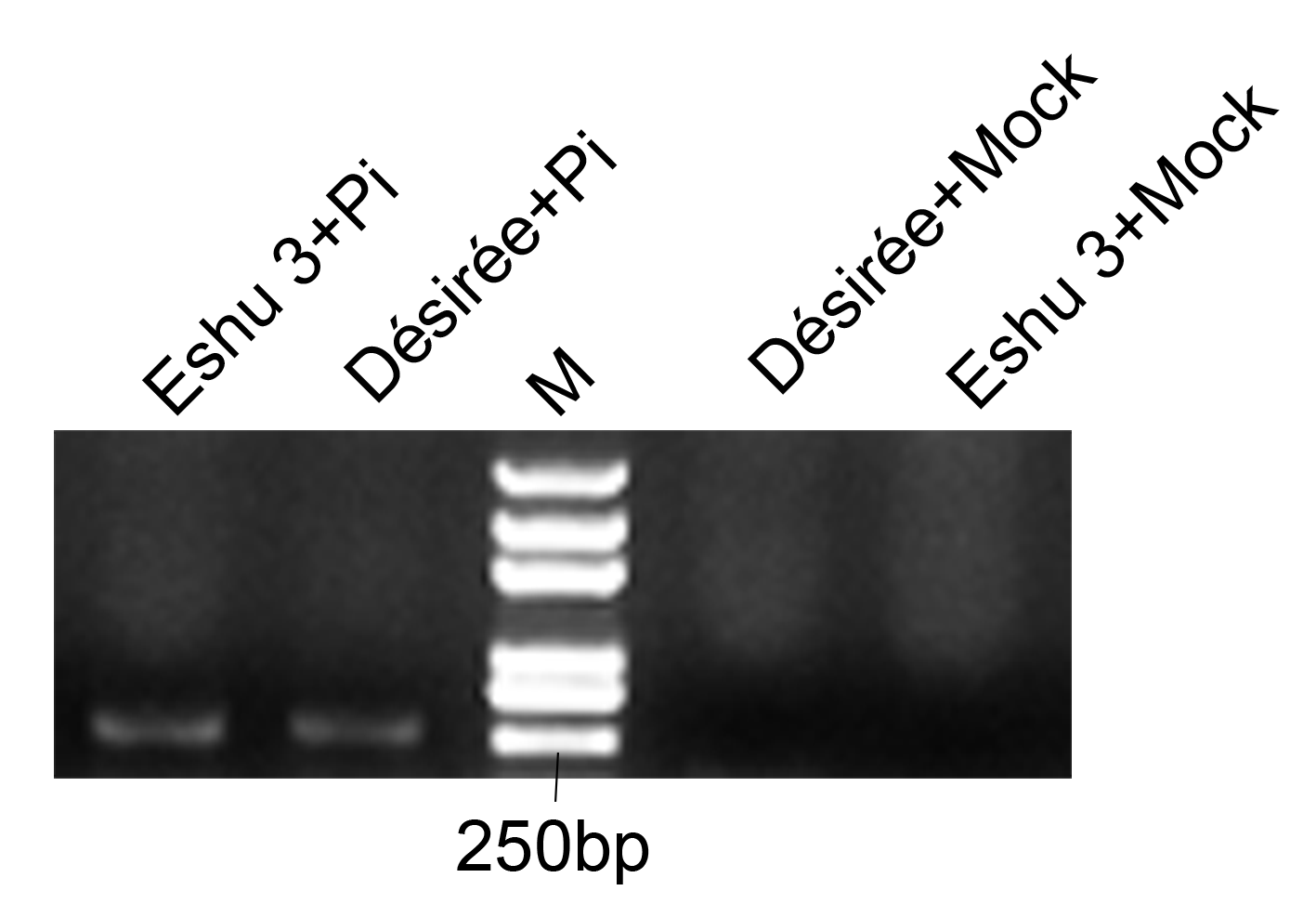

Supplement: Supplementary Figure 5 — Identifying the colonization of P. infestans in potato leaves by using RT-PCR. [file Image_5.TIF]
